# The regulatory activities of MALAT1 in the development of bone and cartilage diseases

**DOI:** 10.3389/fendo.2022.1054827

**Published:** 2022-11-14

**Authors:** Di Zhang, Jinhua Xue, Fang Peng

**Affiliations:** ^1^ Department of Medical Imaging, First Affiliated Hospital of Gannan Medical University, Ganzhou, China; ^2^ School of Basic Medicine, Gannan Medical University, Ganzhou, China; ^3^ Department of Pathology, Ganzhou People’s Hospital, Ganzhou, China

**Keywords:** MALAT1, bone, cartilage, ECM degradation, osteoblasts, chondrocytes

## Abstract

Long non-coding RNAs (lncRNAs) have been comprehensively implicated in various cellular functions by mediating transcriptional or post-transcriptional activities. MALAT1 is involved in the differentiation, proliferation, and apoptosis of multiple cell lines, including BMSCs, osteoblasts, osteoclasts, and chondrocytes. Interestingly, MALAT1 may interact with RNAs or proteins, regulating cellular processes. Recently, MALAT1 has been reported to be associated with the development of bone and cartilage diseases by orchestrating the signaling network. The involvement of MALAT1 in the pathological development of bone and cartilage diseases makes it available to be a potential biomarker for clinical diagnosis or prognosis. Although the potential mechanisms of MALAT1 in mediating the cellular processes of bone and cartilage diseases are still needed for further elucidation, MALAT1 shows great promise for drug development.

## Introduction

Non-coding RNAs (ncRNAs) are commonly divided into four groups, including long ncRNAs (lncRNAs), microRNAs (miRNAs), circular RNAs, and pseudogenes. Biologically, ncRNAs have been implicated in the gene expression regulation at the levels of transcription and post-transcription. LncRNAs, usually more than 200 nucleotides in length, have been demonstrated to be associated with the pathogenesis and progression of various diseases. Due to alternative cleavage and/or pre-mRNA splicing and polyadenylation, many different isoforms can be produced from the same locus by lncRNAs (1). This may lead to generation of multiple and diverse lncRNAs. However, most of lncRNAs are expressed at a low level and restricted to specific sub-cell localizations/cell lines/developmental stages/physiological conditions (2). It can be postulated that lncRNAs are remarkably involved in the physiological and pathological processes.

Metastasis‐associated lung adenocarcinoma transcript‐1 (MALAT1, also known as NEAT2 or HCN), encoded on the chromosome 11q13.1 in human and 19qA in mouse, is a highly conserved lncRNA that is retained in the nucleus and abundantly expressed in various cells (3). Evolutionally, MALAT1 shows about 50% conservation in overall sequence and more than 80% conservation in the triple-helix transcript at 3’ end (4). The expression of MALAT1 is high, and this might be related to the strong activity of the promoter and the stability of the transcribed RNA (5). MALAT1, a well-studied lncRNA in human disease, has been implicated in cell differentiation, proliferation, and death. For example, co-culture of prostate cancer PC3 cells and osteoblasts may up regulate the expression of MALAT1 in PC3 cells, and co-culture with SOST^KO^ osteoblasts may further enhance MALAT1 expression, promoting bone metastasis (6).

Bone undergoes constant modeling and remodeling to maintain homeostasis, which requires multiple regulations in cell differentiation, proliferation, and apoptosis. Disturbance of bone homeostasis may lead to the pathogenesis and progression of bone diseases, such as osteoporosis (OP), osteoarthritis (OA), and rheumatoid arthritis (RA) (7, 8). LncRNAs have been reported to epigenetically regulate the differentiation of mesenchymal stromal cells (MSCs) and the development of diseases (9). It has been reported that the expression of MALAT1 in patients with OP is significantly lower than that in healthy people (10). Bone mineral density (BMD) is a diagnostic factor for OP, and genome-wide association study have identified that the intronic variants at MALAT1 gene locus are associated with low BMD in the Qatari population (11). MALAT1 promotes ossification of the posterior longitudinal ligament (OPLL) by enhancing the transcriptional expression of connexin 43 (Cx43) indirectly and sponging miR-1. Deletion of MALAT1 expression may lead to inhibition of Cx43 expression, osteogenesis, proliferation, and inflammation in OPLL fibroblasts (12). The critical roles of MALAT1 in bone diseases have been demonstrated, and this article provides a review on this field.

## The biological activities of MALAT1

### The biological functions of MALAT1 in splicing

**Figure 1:**
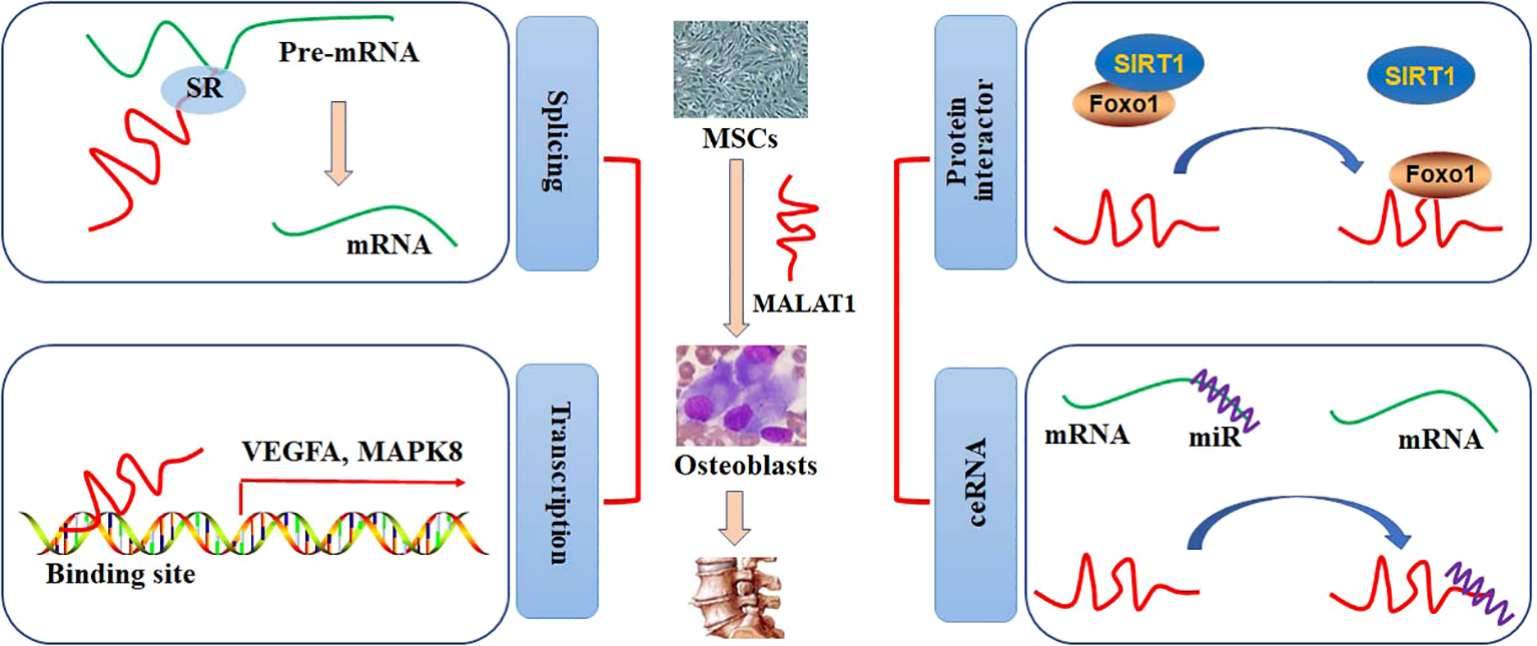
The roles of MALAT1 in bone and cartilage diseases. The regulatory activities of MALAT1 in osteogenic differentiation can be performed by mediating splicing and transcription and acting as a protein interactor or ceRNA.

Nuclear speckles, consisting of RNAs and proteins, are the organelles with self-assembly, governing some specific steps involved in gene expression, such as transcription, splicing, mRNA export. Most of the splicing and transcription are performed at the periphery sites of the speckles, and RNA maturation is modulated at the central sites (13). It has been reported that MALAT1 is abundantly nuclear distribution and located at the periphery sites of the speckles. A study shows that MALAT1 is not essential for the formation of nuclear speckles, and MALAT1 deficiency does not produce any effects on the number, size, distribution, assembly, and structural maintenance of nuclear speckles (14). However, it is demonstrated that depletion of MALAT1 may induce an appropriate 50% reduction of SON expression, which is a scaffold protein in the nuclear speckle. This may, at least partially, result in a decrease in nuclear speckle size in MALAT1-knockout cells (15). Interestingly, MALAT1 tends to interact with many pre-mRNA splicing factors, such as SON1, SRSF1, hnRNPH1, and hnRNPC, which are accumulated in the nuclear speckles. Thus, pre-mRNA splicing can be alternatively regulated by the expression of MALAT1 (16). In addition, MALAT1 may regulate the expression of COL1A1 at the RNA level. Specifically, depletion of MALAT1 can lead to aberrant splicing and decreased distributing sites of COL1A1 RNA (15). Genome-wide transcriptome study shows that MALAT promotes cell proliferation by regulating the pre-mRNA splicing of cell cycle-mediated transcription factor B-MYB in human diploid fibroblasts (17). Dissociation of MALAT1 with the nuclear speckles may also produce negative effects on the transcriptional regulation of target genes (18) (**Figure 1**).

### The biological functions of MALAT1 in transcription

MALAT1 has been shown to account for a high percentage of the chromatin fraction and be actively associated with transcriptional gene expression. In MCF-7 cells, MALAT1 can preferably interact with the active genomic sites. For example, MALAT1 physically binds to CTCF sites and increases the activity of the promoters (19). Spatially proximal to the target binding sites may increase the binding affinity to lncRNAs than distant binding sites. MALAT1, locates at the upstream (60 Kb approximately) of LTPB3 promoter, can be recruited to the promoter of LTPB3 with higher scores, indicating either RNA-DNA or RNA-protein-DNA interaction (19). Chromatin immunoprecipitation (ChIP) has been employed to identify that MALAT1 can regulate the gene expression of EEF1A1 in MDA-MB231 and SKRB3 cells by binding to the regulatory elements in the promoter. Knockdown of MALAT1 expression may abolish cell proliferation and invasion (20). Clinically, highly expression of MALAT1 is positively correlated with poor overall survival in patients with phase III and IV GC. However, no statistical difference is observed in patients with phase I and II GC (21). The association between MALAT1 and EZH2 may provide a novel therapeutic strategy for management of many cancers, such as GC and prostate cancer (22). MALAT1, acts as a chromatin associated RNA, binds to the target genomic sequences by proximity ligation. By employing new technologies, such as mapping RNA-genome interactions (MARGI) and global RNA interaction with DNA by deep sequencing (GRID-seq), a large body of genomic loci for MALAT1 binding has been explored (23, 24). The diagnostic and prognostic effects of MALAT1 in patients with pancreatic cancer have been evaluated by Gene Expression Omnibus, Oncomine, and The Cancer Genome Atlas databases. CCND1, MAPK8, VEGFA, FOS, CDH1, and HSP90AA1 have been identified as the target genes of MALAT1 in patients with pancreatic cancer (25) (**Figure 1**).

### The biological functions of MALAT1 as a protein interactor

The regulatory activity of MALAT1 at the transcriptional level has been demonstrated by binding to the specific sites in the chromatin/genome or interacting with the transcriptional factors or co-activators. In mice diabetic kidney, high glucose-increased expression of MALAT1 is positively related to the levels of serum creatinine and urinary albumin. Further study shows that MALAT1 down regulates the expression of SIRT1 by competitively binding to Foxo1 and disrupting the interaction between SIRT1 and Foxo1 in HK-2 cells (26). In lung transplant ischemia-reperfusion (LTIR) rats, enhanced expression of MALAT1 may up regulate IL-8 expression by recruiting p300, ameliorating inflammatory responses. Silencing of MALAT1 expression can reversely inhibit inflammatory injury in LTIR rats (27). MALAT1 may associate with EZH2, a subunit catalyzing the trimethylation modification in histone 3 Lys27 (H3K27me3), to inhibit the expression of tumor suppressor PCH10 and increase migration and invasion in gastric cancer (GC) cells (21). MALAT1/EZH2 can also negatively inhibits KEAP1 transcriptional expression, leading to up regulation of NRF2 expression in multiple myeloma cells (28). In a high throughput experiment, 127 potential proteins are identified as the interacting targets of MALAT1. Further study shows that MALAT1 may competitively interact with DBC1, resulting in SIRT1 release, p53 deacetylation, cell proliferation, and inhibition of cell apoptosis (29). The roles of MALAT1 in cancer cells have been reviewed (30). Knockdown or overexpression of MALAT1 under different pathophysiological conditions exhibits substantial transcriptional changes (**Figure 1**).

### The biological functions of MALAT1 as a competing endogenous RNA

A large body of studies report MALAT1 as a miRNA sponge or a ceRNA in various cell lines. In high glucose-treated HK-2 cells, up regulated expression of MALAT1 significantly inhibits cell viability, increases apoptosis, and promotes the productions of IL-1β, IL-6, and TNFα by sponging miR-16b-5p, which targets to degrade TLR4. Knockdown of MALAT1 expression alleviates high glucose-induced injury in HK-2 cells, protecting against diabetic nephropathy (31). In human liver tissues, the up regulation of MALAT1 expression is associated with the progression of nonalcoholic fatty liver disease (NAFLD). In fatty acid-treated hepatocytes *in vitro*, knockdown expression of MALAT1 ameliorates the inhibitory activity against miR-206, which degrades ARNT. As a transcriptional factor, ARNT can bind to and inhibit the promoter activity of PPARγ. Thus, MALAT1/miR-206/PPARγ can be a therapeutic target against NAFLD (32). Similarly, MALAT1 acts as a sponge to inhibit miR-181a, which is up regulated in angiotensin II-treated cardiomyocytes. Decreased expression of MALAT1 promotes miR-181a-induced cardiac hypertrophy by decreasing the expression of HMGB2 (33). By sequestering miRNAs, MALAT1 may directly/indirectly regulate the pathophysiological processes of many diseases, providing a potential target for clinically therapeutic management (**Figure 1**).

## The roles of MALAT1 in osteoporosis

### The regulatory mechanism of MALAT1 in osteogenic differentiation

Osteogenic differentiation of bone marrow-derived MSCs (BMSCs) is critical for the formation of bone. Both osteoblasts and osteoclasts are differentiated from BMSCs. The complex regulatory mechanisms orchestrate the processes of osteogenic differentiation (**Figure 1**). It has been reviewed that many signaling pathways, such as Wnt/β-catenin, TGF-β1/Smad2/3, BMP, and PI3K/AKT, have been involved in the regulation of osteogenic differentiation (34). The importance of lncRNAs in gene regulation at the epigenetic, transcriptional, and post-transcriptional levels has been identified in the stages of osteogenic differentiation. These regulations include histone modification, transcriptional interference, nuclear and cytoplasmic trafficking, genomic imprinting, and X-chromosome inactivation (35). One study has been shown that 89 lncRNAs from the constructed lncRNA-miRNA-mRNA network are differentially expressed after osteogenic differentiation, mediating the expression of Wnt/β-catenin, TGF-β1/Smad2/3, and PI3K/AKT signaling pathways in dental pulp stem cells (36). Furthermore, the lncRNAs-transcription factor (TF) feedback loops are identified. Specifically, lncRNAs and TFs may compose feedback loops to mediate osteoblast differentiation, due to that TFs can directly mediate the differentiation activity by binding to the DNA-regulatory elements of lncRNAs (37).

MALAT1 has been considered as a biomarker in OP and is related to osteogenic differentiation (38). During the culture period of human MSCs, the osteogenic differentiation is developed from day 1 to 14. MALAT1 promotes the expression of osterix (Osx) and increase osteogenic differentiation by sponging miR-143 in human BMSCs (39). MALAT1 has been reported to increase RUNX2-regulated osteogenic differentiation of adipose-derived MSC (ADSCs) by specifically sponging miR-30 (40). In osteogenic hBMSCs, the expression of MALAT1 and Osx is up regulated, and the expression of miR-96 is down regulated (41). Co-culture of BMSCs-derived exosome and human osteoblasts increases SATB2-mediated osteoblast differentiation and proliferation by enhancing MALAT1 expression and attenuating miR-34c expression. Up regulation of MALAT1 expression can ameliorate the pathological changes of OP in ovariectomized (OVX) mice (42).

Simultaneously, the expression of MALAT1 is significantly increased. Knockdown of MALAT1 expression can attenuate osteogenic differentiation by mediating has-miR-214-3p/BMP2 axis (43) (**Table 1**). In microgravity-treated MC3T3-E1 cells, the expression of MALAT1 is down regulated, accompanied by decreased expression of alkaline phosphatase (ALP), Col1a1, and BMP4. In human aortic valve interstitial cells (hAVICs), MALAT1 functions as a positive mediator in osteogenic differentiation through Smad4 by suppressing the expression of miR-204 (**Table 1**), as indicated by increased expression of ALP and osteocalcin (OCN) and mineralization (44). Overexpression of MALAT1 can promote osteogenic differentiation by regulating the activity of miR-127/AKT3 axis, alleviating OP (38). Consistently, the expression of MALAT1 is up regulated after osteogenic differentiation of BMSCs and down regulated after osteoclast differentiation of mononuclear macrophages. Knockdown of MALAT1 expression suppresses osteogenic differentiation and facilitates osteoclast differentiation by mediating miR-124-3p/IGF2BP1 axis (**Table 1**). Overexpressed MALAT1 stimulates the activity of Wnt/β-catenin signaling pathway, ameliorating bone injury in mice (10).

**Table 1:** MALAT1 promotes osteogenic differentiation by activating transcriptional factors (TFs) through interacting with miRNAs.

Cell types	miRNAs	TFs	Osteogenic effects	Ref.
hMSCs	has-miR-214-3p	BMP2	Runx2↑, ALP↑, Alizarin Red S↑	(43)
hBMSCs	miR-143	Osx	ALP↑, OCN↑, OPN↑, Alizarin Red S↑	(39)
ADSCs	miR-30	Runx2	ALP↑, OCN↑, OPN↑, Osx↑	(40)
hBMSCs	miR-96	Osx	ALP↑, Alizarin Red S↑	(41)
hAVICs	miR-204	Smad4	ALP↑, OCN↑, Alizarin Red S↑	(44)
hPLSCs	miR-155-5p	ETS1	Runx2↑, ALP↑, OCN↑, Alizarin Red S↑, collagen-1↑	(45)
hBMSCs	miR-34c	SATB2	ALP↑, Alizarin Red S↑, Runx2↑, ATF4↑, Hoxa2↓	(42)
BMSCs	miR-124-3p	IGF2BP1	ALP↑, Alizarin Red S↑, Wnt/β-catenin↑	(10)
MC3T3-E1 cells	miR-217	AKT3	ALP↑, BMP4↑, Col1a1↑, Spp1↑	(38)

↑ means up regulation, and ↓ is down regulation.

In a study, the expression of MALAT1 in rat OP models is down regulated. However, it is also shown that MALAT1 can induce inhibition of osteogenic differentiation by increasing the activity of p38 MAPK and ERK1/2 signaling pathways in BMSCs (46). This conclusion seems to be contradictory, due to the confusion by the different cell lines and experimental conditions. It has been reported that p38 MAPK is essential for bone formation and homeostasis in stem cells (47). Activation of p38 MAPK and ERK1/2 pathways increases the expression of RUNX2 and Osx, leading to enhancement of osteogenic differentiation (48, 49). MALAT1 deletion may ameliorate RANKL-induced inhibition of cell growth and arrest of cell cycle in hFOB 1.19 osteoblast cells (50). Consistently, MALAT1 overexpression is associated with OPG down regulation and RANKL up regulation, respectively, promoting osteoclast processes in ultra-high molecular weight polyethylene (UHMWPE)-treated hFOB 1.19 cells. Potentially, MALAT1 may increase the expression of VEGF by sponging miR-22-5p, promoting the onset of osteolysis (51).

### The clinical potentials of MALAT1 against OP

Osteogenic differentiation of MSCs may promote bone regeneration, which involves multiple-step- and multiple-gene-mediated processes that are implicated in the recovery of bone diseases, such as OP and bone fracture. By using RNA-seq technology, 1524 differential expressed lncRNAs have been identified in osteo-inductive groups compared with the control group. The potential lncRNA-miRNA-mRNA network in rat BMMSCs has been explored (52). Many lncRNAs, such as MALAT1, H19, and HOTAIR, have been summarized to be associated with bone regeneration and balance of bone formation and resorption (53). Several reviews focusing on the contribution of MALAT1 to osteogenesis and endochondral ossification have been explored (54). In the exosomes from BMSCs in patients with postmenopausal OP, 148 lncRNAs are up regulated, and 138 are down regulated. Analysis of constructed lncRNA-miRNA-mRNA network indicates that these differential expression of lncRNAs may potentially associate with Wnt/β-catenin, PI3K/AKT, and MAPK signaling pathways (55). A total of 1878 differential expressed lncRNAs from patients with steroid-induced osteonecrosis of the femoral head have been detected using microarray and bioinformatics. MALAT1 is one of the ceRNA relating to abnormal osteogenic differentiation of BMSCs (56).

In steroid-induced avascular necrosis of the femoral head, the expression of MALAT1 is decreased. MALAT1 overexpression or miR-214 inhibition can ameliorate dexamethasone (DEX)-induced inhibition of osteogenic differentiation of BMSCs (57). The expression of MALAT1 in pre-osteoblast MC3T3-E1 cells can be down regulated by DEX, which has been demonstrated to inhibit osteoblast proliferation and promote osteoblast apoptosis (58). More specifically, forced expression of MALAT1 can abrogate DEX-induced viability repression and cell apoptosis by suppressing Ppm1e expression and activating AMPK signaling in OB-6 and hFOB1.19 osteoblast cells (59). Promoted osteoblast differentiation, inhibited osteoclast function, and balanced metabolism are important for the treatment of osteoporosis (OP). However, in rats with OP tibial fracture, the expression of MALAT1 is increased. Knockdown of MALAT1 expression can enhance osteogenic differentiation of BMSCs by increasing the expression of miR-144-3p (60). This discrepancy might be associated with different cell lines and culture conditions. Bone repair processes include bone resorption by activating osteoclastogenesis and bone formation by activating osteogenesis and neovascularization. Endothelial progenitor cells (EPCs)-derived exosomes can promote the migration and osteoclasts differentiation by increasing the expression of MALAT1, providing a therapeutic strategy for bone repair. Specifically, MALAT1 can increase the expression of ITGB1 by sponging miR-124 in EPC-derived exosomes (61).

In RAW 264.7 cells, co-culture of MCF-7 cells may activate osteoclastogenesis. Denosumab, an inhibitor of receptor activator of NF-κB ligand (RANKL), has been reported to inhibit MCF-7 cell-induced osteoclastogenesis by decreasing the expression of MALAT1 (62). In human periodontal ligament stem cells (hPLSCs), MALAT1 increases ALP activity and mineralization, enhances RUNX2, collagen I, and OCN expression, and promotes osteogenic differentiation by mediating miR-155-5p/ETS1 signaling, providing a strategy for treatment of periodontitis (45) (**Table 1**). Bisphosphonates (BPs), have been used for treating bone lesions, including those associated with multiple myeloma (MM). However, it is reported that BPs may potentially produce adverse effects, such as osteonecrosis of the jaw (63). The altered profile of lncRNAs in MM patients with osteonecrosis induced by BPs indicates that the down regulated expression of MALAT1 is assumed to be related to increased osteoclastogenesis in bone lesions (64). Angiogenesis contributes to bone regeneration by connecting with osteocytes. The expression of MALAT1, VEGF, and SP1 are increased in osteogenic medium-stimulated MC3T3-E1 cells. Knockdown of MALAT1 expression inhibits angiogenesis and bone regeneration, as shown by decreased proliferation, migration, and capillary tube formation of human umbilical vein endothelial cells (65).

Collectively, the expression of MALAT1 in OP is decreased, and enhanced expression of MALAT1 may promote osteogenic differentiation and osteoblast proliferation and inhibit osteoclast differentiation and osteoblast cell death.

## The roles of MALAT1 in osteoarthritis

Osteoarthritis (OA) is marked by chronic inflammation, chondrocyte apoptosis, extracellular matrix (ECM) degradation, and articular cartilage destruction, resulting in joint pain and disability. Chondrocytes, the unique cell type in cartilage, maintain the micro-environmental homeostasis. In addition, the synthesis and degradation of ECM are balanced by chondrocytes, which are orchestrated by network of signaling pathways. To provide effective prevention and treatment of OA, it is essential to understand the complex pathogenesis and pathophysiological processes. It has been demonstrated that lncRNAs are closely associated with the pathogenesis and progression of OA (66). Bioinformatics analysis reveals that 3007 lncRNAs are up regulated and 1707 lncRNAs are down regulated in OA chondrocytes. Additionally, 530 lncRNAs have been found to regulate their target genes expression by interacting with TF SP1 (67). Under IL-1β stimulation, 125 differentially expressed lncRNAs are identified and act as important regulator in the inflammatory responses in OA cartilage (68). MSCs with the capability of self-renew may promote tissue repairment. Injection of MSCs into the articular cavity can significantly impede the degradation of cartilage and improve joint biological functions (69). Extracellular vesicles (EVs) secreted from MSCs have been reported to ameliorate the pathological alternations and inflammation in cartilage. Specifically, EVs with MALAT1 overexpression inhibit the expression of MMP-13, IL-6, and caspase-3 in IL-1β-treated C28/I2 cells, promoting chondrocyte proliferation and migration and compromising inflammation (70).

In patients with OA, the expression of MALAT1 is increased. In LPS-treated rat chondrocytes, silenced expression of MALAT1 increases Collagen II expression and decreases MMP-13, IL-6, and COX-2 expression, protecting ECM against LPS-induced degradation. Mechanically, MALAT1 plays a critical role in LPS-induced pathological changes in chondrocytes by activation PI3K/AKT/mTOR signaling pathway *via* sponging miR-146a (71). Similarly, MALAT1 also promotes ECM degradation by activating the expression of a disintegrin and metalloproteinase with thrombospondin motifs-5 (ADAMTS-5) by binding to miR-145 in human primary chondrocytes *in vivo* and *in vitro*. Knockdown of MALAT1 expression may contribute to cell viability and the stability of ECM in IL-1β-treated chondrocytes (72). MALAT1 may activate the expression of NF-κB signaling by interacting with miR-9, increasing the productions of IL-6, MMP-13, and caspase-3 in mice chondrocytes. Resveratrol has been demonstrated to bind to the promoter of MALAT1 and inhibit its transcriptional expression, therefore inhibiting NF-κB signaling-mediated inflammation, ECM degradation, and chondrocyte apoptosis (73).

Conversely, excessive proliferation may contribute to the pathological alternations of OA. A study shows that MALAT1 may enhance cell proliferation, inhibit cell apoptosis, and ameliorate the degradation of ECM in IL-1β-treated human chondrocytes by stimulating AKT3 expression *via* sponging miR-150-5p (74). Consistently, MALAT1 knockdown can suppress the proliferation of human OA chondrocytes by binding to miR-127-5p, accompanied by decreased expression of PI3K/AKT signaling-related factors and OPN (75). In IL-1β-treated rat chondrocytes, the expression of MALAT1 is down regulated. PcDNA3.1-MALAT1 transfection may increase the viability of chondrocyte and the expression of collagen II and decrease the apoptotic ratio of chondrocytes and the expression of MMP-13 by inhibiting the activity of p-JNK signaling (76).

Pathological changes in OA subchondral bone tissues are also associated with the early alternations of OA. Trabecular thickening can be developed before the initiation of cartilage degeneration (77). MALAT1 expression in subchondral bone of early OA patients is enhanced, and it is induced by inflammatory stress. Depletion of MALAT1 may result in increased production of PGE2 without affecting OPG synthesis (78). In early stage of OA, synovitis is active. OA patients with synovitis have been found the radiographic signs in cartilage degradation, indicating a potential role of synovitis in accelerating the pathological changes of OA (79). In obese patients with OA, higher expression levels of inflammatory cytokines IL- 6 and CXCL8 in synovial fibroblasts are observed. RNA-seq assays identify that MALAT1 expression is significantly up regulated. Knockdown of MALAT1 expression may compromise the increased expression of inflammatory cytokines and inhibit the proliferation of synovial fibroblasts (80).

Collectively, BMSCs-derived MALAT1 expression contributes to inhibition of inflammation. In OA chondrocytes, the expression of MALAT1 is up regulated and associated with increased inflammation and cell excessive proliferation. Knockdown of MALAT1 in OA chondrocytes may become a potentially therapeutic strategy for clinic management of OA.

## The roles of MALAT1 in intervertebral disc degeneration

Intervertebral disc degeneration (IDD), an aging disease with degenerative characteristics, is often associated with low back pain in clinic. Intervertebral disc (IVD) consists of the nucleus pulposus (NP), annulus fibrosus (AF), and cartilage endplates (CEPs). The pathological changes in NP cells contribute a key role to the development of IDD (81). Differentially expressed lncRNAs from the NP cells of IDD patients have been shown the importance for the pathogenesis and progression of IDD (82). It has been demonstrated that the expression of MALAT1 in NP cells from degenerative IVD is down regulated. Overexpressed MALAT1 may decrease the production of IL-1 and IL6 and inhibit the expression of caspase-3, inhibiting inflammatory responses and cell apoptosis (83). Consistently, down regulated expression of MALAT1 is statistically correlated with decreased production of collagen II and aggrecan in rats with IDD. MALAT1 overexpression exhibits the protective activity against the biological actions produced by IL-1β in NP cells *via* inhibiting the expression of miR-503 and the activation of MAPK signaling pathways (84).

CEPs control the transport and distribution of nutrients in IVDs. Injury in CEPs has been reported to promote IDD development. Diabetes mellitus (DM) contributes the degeneration of CEPs, due to the detrimental effects of high glucose on CEPs by inducing oxidative stress, mitochondrial dysfunctions, and cell apoptosis (85). Controversially, the expression of MALAT1 in high glucose-treated CEP cells is up regulated. Deletion of MALAT1 may decrease CEP cells apoptosis by attenuating the phosphorylation levels of p38 MAPK (86). More efforts are still needed to clearly elucidate the relationship between MALAT1 and diabetic patients with IDD.

## The roles of MALAT1 in rheumatoid arthritis

Rheumatoid arthritis (RA), an autoimmune disease, is marked by chronic inflammation and joint destruction. Till now, no effective and reliable biomarkers for prognosis prediction or therapeutic evaluation are available for RA. The etiological studies suggest that many factors, such as genetic susceptibility, aberrant metabolism, and dysregulated immune actions, are included in the pathogenesis and progression of RA (87). Differentially expressed lncRNAs have been demonstrated to contribute to the development of some auto-immune diseases, such as RA (88). Activation of inflammatory and immune responses and increased angiogenesis in synovial cells have been reported to promote the pathogenesis of RA (89, 90). These biological effects are also involved in the regulation of MALAT1 as discussed above. It is reasonable to postulate that MALAT1 is implicated in RA development.

MALAT1, MEG3, and NEAT1 have been identified as the key potential lncRNAs involved in certain physiological and pathological activity in RA patients. Specifically, the expression of MALAT1 in the peripheral blood mono-nuclear cells (PBMCs), plasma, and synovial fluid of RA patients is significantly up regulated (91). In the fibroblast-like synoviocytes (FLS) derived from RA patients, deletion of MALAT1 can decrease cell apoptosis and activate PI3K/AKT signaling pathway. Quercetin may exhibit the protective activity against MALAT1-induced pathological changes in RA FLS (92). It has been reported that MALAT1 can be considered as a potential biomarker for RA diagnosis, due to being statistically correlating with the expression of HSPA5 (GRP78) and MMD (PAQR1) in patients with RA (93). However, the single nucleotide polymorphism of MALAT1 gene is reported to be not correlated with the susceptibility of RA (94).

## The roles of MALAT1 in juvenile idiopathic arthritis

Juvenile idiopathic arthritis (JIA), a chronic rheumatic disease in children associating with chronic inflammation and inappropriate immune activation, starts before 18 years of age and lasts at least 6 months, according to the updated classification criteria (95). Drugs available for children with JIA are rather limited (96). LncRNAs have been shown to regulate immune abnormalities and the development of JIA. For example, lncRNA RP11-340f14.6 can bind to the neighbor P2X7R and increase its expression in patients with JIA. In addition, RP11-340f14.6 up regulates the differentiation of Th17 and down regulates Treg distribution in a P2X7R-independent manner (97). In patients with systemic JIA, the expression of MALAT1 is significantly up regulated, and the productions of IL-6/-17/-1β, TNFα, and MMP-8/-9 in the plasma are also increased. Knockdown of MALAT1 expression may reverse the abnormal levels of these cytokines. MALAT1 ameliorates the progression of systemic JIA by activating JAK/STAT signaling pathway through sponging miR-150-5p (98).

## The roles of MALAT1 in ankylosing spondylitis

Ankylosing spondylitis (AS), a chronic immune-mediated inflammatory disease featured as bone erosion and syndesmophyte formation, mainly occurs in the axial skeleton and the sacroiliac joints. Bone destruction may be related to activation of RANKL system and inactivation of Wnt/β-catenin pathway (99). In addition, many immunocytes, such as Th1/Th2/Th22 cells, dendritic cells, and T/B lymphocytes, have been reported to be aberrantly activated (100). However, the molecular mechanisms underlying the pathogenesis and progression of AS remain unclear, and the clinical therapeutic management of AS is dissatisfying. Evidence shows that lncRNAs play a pivotal role in AS development (101). The expression of MALAT1 is up regulated in cartilage tissue and chondrocytes isolated from AS patients. Decreased expression of MALAT1 enhances cell viability and inhibits pyroptosis by suppressing GSDMD expression and increasing miR-558 expression in AS chondrocytes (102).

## The roles of MALAT1 in gouty arthritis

Hyperuricemia-induced gouty arthritis (GA) is marked by the deposition of monosodium urate (MSU) crystal in joints and activation of immune inflammation. MSU can be recognized by macrophages, and the system of NF-κB/RANKL is activated. Increased tophus in the joint cavity constantly destroys the bone surface and induces osteoclast differentiation by activating RANKL system (103). Urate-lowering is the critical strategy to therapeutic management of hyperuricemia and gouty. However, adverse effects of current available drugs greatly limit their applications (104). LncRNA has been implicated in the pathogenesis of inflammatory arthritis, such as GA (105). Differentially expressed lncRNAs in patients with GA have been reported, and the predicted regulatory pathways are mainly focusing on inflammation and osteoclast differentiation (106). In MSU-treated THP-1 macrophages, MALAT1 expression is up regulated, and the productions of IL-1β, TNFα, caspase-1, and NLRP3 are also enhanced. The total glucosides extracted from paeony (TGP) may significantly ameliorate the abnormal expression of these factors. Overexpression of MALAT1 can abolish the suppressive activity of TGP on MSU-induced inflammation by mediating miR-876-5p/NLRP3 axis in THP-1 macrophages (107). Targeting MALAT1 may become a potential strategy for therapeutically treating GA.

## Conclusion

Extensive evidence shows that lncRNAs have been implicated in the various genomic processes, affecting cell physiological and pathological functions. MALAT1, a well-studied lncRNA, plays a key role in bone and cartilage diseases by regulating the biological processes of osteogenic differentiation, proliferation, and apoptosis. MALAT1 is abundantly located at nuclear speckles, which mediate the transcription, splicing, and mRNA export. Specifically, there is a facilitatory effect for MALAT1 to interact with the targets, such as mRNA or TFs. The interactions between MALAT1/protein and MALAT1/RNA are the essential ways for MALAT1 to regulate the activity in bone and cartilage diseases, including OP, OA, IDD, RA, JIA, AS, and GA. Particularly, MALAT1 may promote osteogenic differentiation in MSCs. It is interesting that decreased expression of MALAT1 is associated with the pathological changes in OA chondrocytes. Conversely, MALAT1 exhibits a stimulating effect on the pathological development of RA, JIA, AS, and GA. However, the molecular mechanisms of MALAT1 in regulating the pathogenesis and progression of bone and cartilage diseases remain largely unclear. More efforts are still needed.

## Author contributions

FP: Conceptualization, Methodology. DZ, JX and FP: Data curation, Writing-original draft preparation, Data curation, Validation, Writing-reviewing and Editing. All authors contributed to the article and approved the submitted version.

## Funding

This study was financially supported by the Science and Technology research project of the Education Department of Jiangxi Province (Grant NO. GJJ211520).

## Conflict of interest

The authors declare that the research was conducted in the absence of any commercial or financial relationships that could be construed as a potential conflict of interest.

## Publisher’s note

All claims expressed in this article are solely those of the authors and do not necessarily represent those of their affiliated organizations, or those of the publisher, the editors and the reviewers. Any product that may be evaluated in this article, or claim that may be made by its manufacturer, is not guaranteed or endorsed by the publisher.
